# Gating Patterns to Proprioceptive Stimulation in Various Cortical Areas: An MEG Study in Children and Adults using Spatial ICA

**DOI:** 10.1093/cercor/bhaa306

**Published:** 2020-11-03

**Authors:** Jaakko Vallinoja, Julia Jaatela, Timo Nurmi, Harri Piitulainen

**Affiliations:** Department of Neuroscience and Biomedical Engineering, Aalto University School of Science, 00076 Espoo, Finland; Department of Neuroscience and Biomedical Engineering, Aalto University School of Science, 00076 Espoo, Finland; Department of Neuroscience and Biomedical Engineering, Aalto University School of Science, 00076 Espoo, Finland; Faculty of Sport and Health Sciences, University of Jyväskylä, FI-40014 Jyväskylä, Finland; Department of Neuroscience and Biomedical Engineering, Aalto University School of Science, 00076 Espoo, Finland; Faculty of Sport and Health Sciences, University of Jyväskylä, FI-40014 Jyväskylä, Finland; Aalto NeuroImaging, MEG Core, Aalto University School of Science, 00076 Espoo, Finland

**Keywords:** independent component analysis, magnetoencephalography, paired stimulus, proprioception, somatosensory

## Abstract

Proprioceptive paired-stimulus paradigm was used for 30 children (10–17 years) and 21 adult (25–45 years) volunteers in magnetoencephalography (MEG). Their right index finger was moved twice with 500-ms interval every 4 ± 25 s (repeated 100 times) using a pneumatic-movement actuator. Spatial-independent component analysis (ICA) was applied to identify stimulus-related components from MEG cortical responses. Clustering was used to identify spatiotemporally consistent components across subjects. We found a consistent primary response in the primary somatosensory (SI) cortex with similar gating ratios of 0.72 and 0.69 for the children and adults, respectively. Secondary responses with similar transient gating behavior were centered bilaterally in proximity of the lateral sulcus. Delayed and prolonged responses with strong gating were found in the frontal and parietal cortices possibly corresponding to larger processing network of somatosensory afference. No significant correlation between age and gating ratio was found. We confirmed that cortical gating to proprioceptive stimuli is comparable to other somatosensory and auditory domains, and between children and adults. Gating occurred broadly beyond SI cortex. Spatial ICA revealed several consistent response patterns in various cortical regions which would have been challenging to detect with more commonly applied equivalent current dipole or distributed source estimates.

## Introduction

Sensory gating of repeated stimuli is a robust well-demonstrated phenomenon, where successive sensory stimuli presented with short intervals result in the reduction of the respective response amplitudes at the sensory cortices (Fruhstorfer et al. 1970; McLaughlin and Kelly 1993). Maximum responses are achieved only at interstimulus interval (ISI) of several seconds or even tens of seconds, depending on the response latency, cortical area, and sensory modality. For example, the primary cortical evoked fields are attenuated at ISIs below 8–16 s for auditory (Hari et al. 1982; Lü et al. 1992; Forss et al. 1993), below 1 s for visual (Uusitalo et al. 1997) and below 8 s for proprioceptive (Smeds et al. 2017) stimuli. The exact neuronal mechanisms of the sensory gating are not known, but it is suggested to be important to prevent excessive afference and thus overloading the sensory processing resources in the brain (Lundstrom et al. 2008; Wang, Webber, et al. 2010). One possibility is that the gating in the primary sensory cortices is caused by an inhibitory effect from related higher level cortical processes. It has been proposed that frontal networks may modulate task relevant gating (Knight et al. 1999; Staines et al. 2002). In the auditory domain, it indeed appears that the prefrontal cortices may regulate the sensory gating (Mayer et al. 2009).

Paired-stimulus paradigm has been used to study gating in a controlled quantifiable manner. Majority of the studies have focused on auditory domain, presenting brief clicks or beeps, or somatosensory domain using electrical stimulation of the median or tibial nerve. There are several works using tactile stimuli (Wühle et al. 2011; Spooner et al. 2019) and some using passive movements (Angel et al. 1985), but we still lack a large body of literature about gating in variety of stimulus modalities. In addition, the development of gating phenomenon is still poorly understood.

Sensory gating clearly has a functional relevance. The best demonstrated examples are from schizophrenia patients who demonstrate reduced auditory gating (e.g., Adler et al. 1982; Boutros et al. 1991; Freedman et al. 1991; Budnick and Braff 1992; Adler et al. 1998; Boutros et al. 1999). Gating alterations can be specific for a given modality and cortical area. For example, in schizophrenia, somatosensory gating in the primary somatosensory (SI) cortex shows reciprocal effects to the auditory gating, whereas the secondary somatosensory (SII) cortex shows coaxial effects with the auditory gating (Edgar et al. 2005; Thoma et al. 2007). Somatosensory gating has been observed to decline in aging population (Cheng and Lin 2013; Spooner et al. 2019). Alterations have also been observed in some motor disorders. For example, children with cerebral palsy exhibit hyper-gating to tibial nerve stimulation (Kurz et al. 2018).

One of the most fundamental somatosensory senses for smooth motor performance is the proprioception (“movement sense”). The proprioceptors are located in muscles and joints and are sensing limb positions, movements, and forces (for a review, see Proske and Gandevia 2012). The proprioceptive afference to the brain is integrated with the vestibular system to provide the brain with information about the internal state of the locomotor system and its spatial orientation. Behavioral proprioceptive perception is often impaired in movement disorders such as Parkinson’s (Konczak et al. 2007), dystonia (Avanzino and Fiorio 2014), chorea (Sharp et al. 1994), and cerebral palsy (Wingert et al. 2009). Precise computer-controlled movement actuators can be used to stimulate the proprioceptors in order to study the cortical proprioceptive processing using magnetoencephalography (MEG; Alary et al. 2002; Lange et al. 2001; Piitulainen et al. 2015; Piitulainen, Seipäjärvi, et al. 2018b) or fMRI (Nurmi et al. 2018). In MEG, the cortical responses to passive movements primarily reflect the processing of proprioceptive afference with negligible effect of the tactile afference (Piitulainen et al. 2013; Bourguignon et al. 2015). Therefore, the evoked movements are feasible to examine the proprioceptive gating when using MEG, with good reproducibility (Piitulainen, Illman, et al. 2018a).

The majority of the direct proprioceptive inputs to the cortex via thalamus are directed to the SI cortex, but some also the primary motor (M1) cortex (Goldring and Ratcheson 1972), and with lesser extent to various other cortical loci involved in sensorimotor processing. In addition, proprioceptive afference activates several cortices indirectly, for example, trough cortico-cortical pathways. When recorded with MEG, the most prominent cortical proprioceptive response is seen in the SI cortex and less prominently in the SII cortex (Alary et al. 2002; Smeds et al. 2017). Beyond these somatosensory cortices, the proprioceptive information is processed within a larger cortical network, including premotor and posterior parietal cortices (e.g., Freund 2003; Schwoebel and Coslett 2005; Pellijeff et al. 2006; Tsakiris et al. 2007). In addition, superior parietal lobule and supramarginal gyrus have also been linked to haptic shape recognition which demands accurate proprioceptive processing of the hand (Miquée et al. 2008).

Separation of distinct neural MEG signal among simultaneously active cortical regions faces several challenges. The cortical extent of somatosensory gating to median nerve stimulation has been attempted to examine using MEG and minimum-norm estimation (Hsiao et al. 2013). The fundamental issue in MEG source separation is that the source time-courses of the brain activity inevitably mix spatially in the recorded MEG signals and are not adequately separated when using, for example, the minimum-norm (Hämäläinen and Ilmoniemi 1994; Dale et al. 2000; Pascual-Marqui 2002; Hauk et al. 2011) or minimum-variance (Van Veen et al. 1997; Sekihara et al. 2001) filter-based MEG inverse methods. Thus, the estimated time-course in each cortical source point is a mixture of the true sources. Therefore, the more subtle activities beyond the dominant widespread SI and SII cortex sources have been challenging to identify, which narrow down the physiological interpretations. Another challenge is interindividual anatomical and functional variation when averaging the cortical activations across individuals, especially without prior knowledge of these neuroanatomical differences. That is, regional specificity is lost if the cortical response location to a given stimulus varies between individuals. Approaches using multiple equivalent current dipole modeling (Sarvas 1987; Mosher et al. 1992) can extract spatially separate MEG signals but require prior knowledge of the response such as the number of significant true sources and preferably also their expected locations and primary current directions. Complex and correlated source distributions that are expected when using naturalistic stimuli would violate these assumptions of simple equivalent current dipoles.

In this work, we aimed to examine the cortical proprioceptive gating to paired evoked-movement stimuli using an independent component analysis (ICA) approach to determine several stimulus-relevant source locations and time-courses from MEG signals. ICA is a well-established data driven method for factoring multiple measurements of mixed signals to statistically independent sources (Hyvärinen and Oja 2000). ICA has been used in M/EEG analysis for several purposes. However, typically the approach has been to extract independent sources by maximizing the temporal sparsity of the components, whereas in this work, we focused on the spatial independence. Spatial ICA is often used in functional MRI analysis (McKeown et al. 1998; Calhoun et al. 2001), but in MEG, it has until now only been used to examine oscillatory cortical activity (Ramkumar et al. 2014). Several methods to generalize ICA to group level analysis have been proposed (for a review, see Calhoun et al. 2009). In this work, we computed first the independent components for each individual separately, and then clustered them across the individuals using a spatiotemporal similarity metric.

Our primary aim was to decompose the proprioceptive gating to several distinct cortical components within and beyond SI cortex, and thus further elucidate the neuronal mechanisms behind the gating phenomenon, sensorimotor integration, and proprioception. We aimed to test the feasibility of the spatial ICA to separate evoked MEG responses to independent components using two different study groups: healthy 1) adults and 2) children and adolescents. Our secondary aim was to examine whether the cortical gating of proprioceptive stimuli is matured in the primary sensorimotor cortex to the adult level in the 10- to 17-year-old children and adolescents.

**Figure 1:**
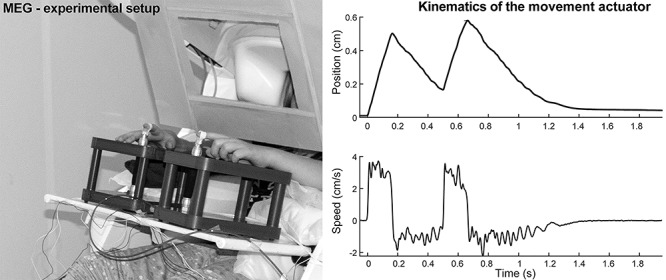
**Left**: The experimental setup. The movement actuators were on table in front of the participant, and a cardboard screen was used to cover the visual contact to the moving finger while allowing viewing the video. **Right**: The displacement and speed of the pneumatic actuator during one stimulus pair measured with a laser distance meter without a finger attached to the device. Onset of the movement is at 0 s.

## Materials and Methods

### Participants

We measured two groups of participants, one consisting of children and adolescents and one of adults. The “child” group included a total of 30 healthy right-handed 10- to 17-year-old children and adolescents who were recruited from school visits, colleague’s family members, and acquaintances. Five participants were excluded from the final data due to data quality issues, which resulted in total of 25 participants in the final analysis, 12 male (48%), mean age: 13.4, SD: 2.3 years. The “adult” group consisted of 21 healthy right-handed adults, 13 male (62%), mean age: 30, SD: 5 years. This group was recruited from faculty employees, friends, and acquaintances, and a pool of volunteers who had indicated their willingness to participate in the faculty research projects. Handedness was determined using the Edinburgh handedness test (Oldfield 1971).

The study was approved by the ethics committee of Helsinki University Hospital (HUS/2318/2016) and was conducted in accordance with the Helsinki declaration. All the volunteers and their guardians gave a written informed consent prior to participation to the study.

### Experimental Setup

A custom-made nonmagnetic pneumatic movement actuator (Aalto NeuroImaging, Aalto University) was used to generate passive right and left index finger flexion extension movements of the metacarpophalangeal joint (Fig. 1). For the adults, only the dominant right-hand stimuli were delivered. For detailed description of the movement actuator, see Piitulainen et al. (2015). In brief, the pneumatic muscle (DMSP-10-100 AM-CM, Festo AG & Co) of the actuator extended ∼5 mm when the pressure inside was quickly dropped from 4 bars to 1 bar evoking an extension of the index finger that was taped on the vertical pneumatic muscle. The participants were instructed to sit as relaxed as possible while watching an uneventful video with slowly moving landscape images. To ensure complete masking of the noises from the movement actuator, the subjects wore earplugs and a constant Brownian noise was played from a panel speaker (Sound Shower, Panphonics). To minimize tactile stimulation during the evoked movements, a layer of surgical tape was used to cover the fingertips of the index fingers. A cardboard screen was used to prevent the participant from seeing the moving finger.

The paired stimulus consisted of two rapid successive extension (upward) movements of the index finger with a 500-ms interval, and with 4000 ± 250 ms interval between the repeated paired stimuli. The 4000 ± 250 ms ISI was chosen because the gating effect was estimated to be reduced close to initial level within 4 s according to Smeds et al. (2017) and to remain within comfortable session duration. Smeds et al. (2017) used the same movement actuator as was used in the current study. The study setup for children included index finger stimulation for both hands and, for adults, the stimulation of right index finger and right ankle. In current work, we only look at responses from right index finger stimulation which was the same for both groups. The stimulus order between the two stimuli was randomized. The recording lasted 14 min totaling at least 100 index finger stimuli. For children, there was a small pause in the middle of the measurement, but the participants remained in the same position during the pause.

### Measurements

MEG recordings were conducted at the MEG Core, Aalto NeuroImaging, Aalto University using a whole-scalp 306-channel (204 gradiometers, 102 magnetometers) MEG system (Vectorview™, Elekta Oy) inside a three-layer magnetically shielded room (Imedco AG) to reduce the external interference. Head position and movement was continuously recorded using five head position indicator coils attached to the head (Fastrak, Polhemus). Prior to the MEG measurement positions of fiducial points, the head position coils, and 200 scalp surface points were registered. Electrooculogram signal was recorded using a pair of electrodes placed below and above the left eye.

For a part of the subjects, the index finger acceleration was recorded simultaneously with MEG signals using a three-axis accelerometer (ADXL335 iMEMS Accelerometer, Analog Devices Inc.) attached on the nail of the moved finger. Acceleration was low-pass filtered at 330 Hz and sampled at 1 kHz, time-locked to the MEG signals. Accelerometer measurements were done to ensure that there were no meaningful differences in the finger movement between participants, although the method has been proved very consistent (Piitulainen et al. 2015). A laser distance meter was used to quantify the distance of the evoked movements in a separate session (Fig. 1).

High-resolution structural *T*_1_-weighted MRI volumes were scanned for each participant (MP-RAGE, Slice thickness: 1 mm, in-plane resolution: 1 mm × 1 mm, TR: 2530 ms, TE: 3*.*30 ms) using a 3 T MAGNETOM Skyra MR scanner (Siemens Healthcare) with a 32-channel head coil. The structural MRI data were co-registered to common coordinates with MEG data using facial features, scalp surface, and fiducial points. The co-registration was done by manually, using tools in MNE-python package (versions 0.16.0 to 0.19.0 were used during this work) (Gramfort et al. 2013), adjusting the coordinate transformation until the points visually matched to facial and scalp features.

### MEG Preprocessing

MEG data were first denoised using oversampled temporal projection (OTP, Larson and Taulu 2017). OTP method assumes that the data are spatially oversampled and reconstruct each sensor data using the other sensors. This means that sensor-specific uncorrelated noise and artifacts are effectively removed. After OTP denoising temporal signal space separation (tSSS, Elekta Maxfilter™, Taulu and Simola 2006) with head movement correction was used to suppress external interference and to correct for the head movement.

FastICA (Hyvärinen and Oja 1997) was used to separate ocular artifacts from the data. We computed 30 components, and the artifact components were chosen by correlating with EOG reference electrode measurement. While tSSS had removed majority of the cardiac activity from the MEG signal, some residual was found with ICA, and those components were also removed. Overall number of removed components for each subject was 1–4 with 3 being the most common number. All removed components were verified manually.

MEG data were band pass filtered between 1 and 40 Hz and downsampled to 100 Hz. The data were divided to baseline corrected 1.7-s epochs (−0.5 to 1.2 s with respect to the onset of the first stimulus of each paired stimulus). Epoch was rejected if signal exceeded the thresholds of 4000 × 10^−13^ T*/*m and 4 × 10^−12^ T for gradiometers and magnetometers, respectively. Each participant’s data were time shifted so that the cortical response onset was at zero. This was done to equalize the differences in the peripheral conduction of proprioceptive afference between participants who were at various stages of growth and development. The onset was determined as the rising edge of stimulus locked activity. To standardize the amount of data between the participants, a random permutation of 90 valid epochs was chosen for each participant.

### MEG Source Analysis

Freesurfer software v. 6.0 (Dale et al. 1999; Fischl et al. 1999) was used to construct a cortical surface model for each participant using the structural MRI scans. MNE software (Gramfort et al. 2014) was then used to create a surface-based source space with 8196 source vertices (4096 for each hemisphere) with average of less than 5-mm distance between them. Each vertex represents a point with three orthogonal dipoles. MNE software was used to create the MEG forward solutions for the source spaces.

The source activity estimation was done using the well-known dSPM method (Dale et al. 2000) which is a minimum norm estimate normalized with prestimulus noise covariance estimates. The MNE-python implementation of the algorithm was used. We used both gradiometers and magnetometers for the source estimation using measured noise covariance to spatially whiten the data for combining sensor modalities. The prestimulus noise covariance was estimated, using shrunk cross-validated covariance estimator, from 0*.*5-s baseline period before the first stimulus control pulse in each of the paired-stimulus events. A minimum norm approach was chosen to minimize a priori assumptions on the nature of the source distributions. The proprioceptive stimuli are expected to produce correlated activity in multiple cortical regions which would have been problematic for a minimum variance beamformer solution. The inverse solution had a depth weighting of 0.8, and dipole orientation was loosely restricted to surface normal (weighting parameter 0.2). Source estimation was done without averaging the epochs. The entire 3D vector solution was estimated using higher prior weight for the cortical normal direction. This resulted in 3 × 8196 = 24 588 time-courses per subject.

### Component Separation

The tSSS algorithm used in the preprosessing reduces the true rank of our 306 channel MEG measurement to be around 70. ICA behaves badly on rank deficient data, and thus, it was necessary to reduce the data dimensionality. This was obviously beneficial for computational purposes as well. We used truncated singular value decomposition (tSVD) to get 65 time-courses (right singular vectors) and their respective spatial source topographies (left singular vectors) instead of the original 24 588 time-courses in the 8196 source points. A total of 65 components were enough to explain more than 98% of the variance in all participants. It is still an open question whether the data should have been reduced further to remove possible noise components. However, since we are looking for individual source locations, we think that we should try to find as small spatial distributions as possible. Dimensionality reduction would possibly reduce the differences between separate sources. As expected, some secondary components of the unaveraged response signal to the proprioceptive stimulus ware relatively weak compared with the other brain activity and noise components. Therefore, significant data reduction would have risked losing physiologically relevant information from the data. We tested a greater dimensionality reduction, but it did not visibly improve the results. The tSVD process can be presented as the following equation:

(1) \begin{equation*} \boldsymbol{X}=\boldsymbol{U}\boldsymbol{\Sigma } {\boldsymbol{V}}^T+{\boldsymbol{U}}_e{\boldsymbol{\Sigma}}_e{\boldsymbol{V}}_e^T, \end{equation*}

where ***U*** contains 65 orthogonal spatial topographies and ***V*** the corresponding 65 time-courses. ***U*** and ***V*** matrices represent spatial and temporal lower rank subspaces of the data matrix.

The spatial ICA analysis approach that we used roughly follows ideas presented by Calhoun et al. for fMRI resting state data (Calhoun et al. 2001). Jonmohamadi et al. (2014) also did similar analysis to find neural sources from MEG evoked responses but using temporal ICA. The independence of components in Spatial ICA can be understood as minimal systematic spatial overlap between the components. We expected true cortical sources to produce separable source distributions in the cortex as long as there is sufficient difference in either the source location or primary current direction. Sources with highly correlated sensor topographies are still expected to be difficult to separate. We used FastICA (Hyvärinen and Oja 1997) algorithm to estimate the unmixing matrix for 65 independent components. The components are given by

(2) \begin{equation*} {\boldsymbol{S}}_s=\boldsymbol{UW}, \end{equation*}

where ***W*** is the ICA unmixing matrix. The respective average time courses can then be found by using the mixing matrix ***W***^−1^

(3) \begin{equation*} {\boldsymbol{S}}_t={\boldsymbol{W}}^{-1}\boldsymbol{\Sigma} {\boldsymbol{V}}_{\mathrm{ave}}, \end{equation*}

where ***V***_ave_ is the average evoked response projected to the space defined by *U*. The components were scaled such that the peak spatial magnitude (norm of the three vector directions) of each component is 1. Finally, the topographies *S_s_* are transformed to MNI152 template brain (Fonov et al. 2009) for group comparison purposes using MNE-python cortical morphing tools. The temporal components ***S****_t_* and the MNI-transformed spatial components ***S****_s_* were stored for each participant and used for clustering.

### Component Clustering

**Figure 2:**
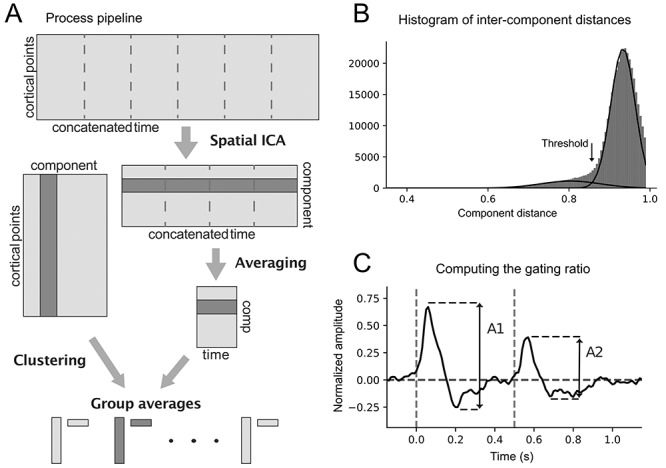
ICA, clustering and gating ratio. (*A*) ICA was performed on concatenated epochs and the temporal averaging was only done after the component separation. Both spatial and temporal information were used on clustering. (*B*) The highest mode of the intercomponent distance histogram represents random similarities and its long-left tail includes the correlations of interest. A Gaussian mixture model (the curves) was used to estimate the threshold for clustering by selecting the point where the histogram becomes more probable as the clustering threshold. (*C*) Peak-to-peak response amplitudes were defined for both stimuli (indicated by the arrows), and the gating ratio was then computed as ratio between them (A2/A1).

Figure 2*A* presents a flow visualization of the ICA-clustering pipeline. We assumed that the components from different participants have spatiotemporal similarities if they represent actual physiological cortical activity related to the proprioceptive stimulation. Thus, we expected to find component clusters across participants that represent physiologically relevant stimulus responses. While dimensionality reduction before ICA might have been problematic, we were able to, at this phase, reject many components that clearly did not present activity that was time locked to the stimulus. We ranked the relevance of components of each participant according to the averaged amplitude within the 0–1.2 s epoch normalized with the prestimulus standard deviation. We then picked 40 most relevant components from each participant and discarded the rest. The number of components was a compromise between data reduction and guaranteeing that all stimulus relevant components were included.

The spatial similarity measure that we applied is simply the Pearson correlation coefficient between the spatial distributions of the components. Notably, we estimated the independent components in 3D vector space but computed the similarities using magnitudes in each 8196 source locations. This choice was made because we were more interested about the approximate location than the exact current dipole direction in the anatomically variable sulci of each participant’s cortex. The similarity of spatial components ***X*** and ***Y*** can then be expressed as

(4) \begin{equation*} S{M}_{s\ \boldsymbol{X},\boldsymbol{Y}}={\rho}_{\boldsymbol{X},\boldsymbol{Y}}. \end{equation*}

The 3D vector components are approximately orthogonal and thus have zero spatial similarity within the participants. However, the source magnitudes of the components are not orthogonal. Thus, two components that have spatially overlapping locations will have nonzero similarity even within the subject.

For temporal similarity, we used the absolute Pearson correlation coefficient between the averaged component time courses. Because some variations in individual timing of the evoked response components between the participants are inevitable, we searched for the strongest correlation with maximum lag of 10 ms between the time courses.

(5) \begin{equation*} S{M}_{t\ \boldsymbol{X},\boldsymbol{Y}}=\underset{\tau }{\max}\left|{\rho}_{\boldsymbol{X}(t),\boldsymbol{Y}\left(t+\tau \right)}\right|,\tau \in \left[-10\mathrm{ms},10\mathrm{ms}\right]. \end{equation*}

We looked for within group clusters that were similar both spatially and temporally. To this end, we used weighted geometric mean of the spatial and temporal similarities to compute the final similarity matrix. Using the geometric mean as compared with arithmetic mean both equalized the effect of spatial and temporal similarity and increased distance between components that are similar only spatially or temporarily and not both. Similarity matrix is

(6) \begin{equation*} \boldsymbol{S}{\boldsymbol{M}}_{st}=\exp \left\{\alpha \times \ln \left(\boldsymbol{S}{\boldsymbol{M}}_s\right)+\left(1-\alpha \right)\times \ln \left(\boldsymbol{S}{\boldsymbol{M}}_t\right)\right\}, \end{equation*}

where *α* ∈ [0*,*1] determines the weighting between spatial and temporal similarity. In this work, we used *α* = 0*.*75 because we aimed to examine rather strictly spatially similar components. However, any other *α* value could be used to reflect the preference between spatial and temporal similarity. We get the dissimilarity matrix.

(7) $$\boldsymbol{D}=\sqrt{1-\boldsymbol{S}{\boldsymbol{M}}_{st}}.$$

The clustering method used was somewhat similar to the one described by Esposito et al. (2005). It is a semi-supervised agglomerative clustering algorithm that adds components one by one to the nearest cluster, ensuring that there is only one component included from each participant. The algorithm that adds components to their nearest cluster provided that the cluster does not already have a component from the same participant, and the distance to all cluster members is below a threshold. Clusters are combined to their nearest neighboring clusters if they do not have components from the same participants and the distance between all the cluster members is below the threshold. This is done until all remaining distances are over the threshold. In the resulting clusters, all intracluster distances are below the threshold. For further analysis, we included only clusters that were well representative of the studied population, that is, clusters with a component from at least two-third of the participants.

The clustering threshold was determined from the distribution of intercomponent distances (Fig. 2*B*). The distribution has a longer left tail that is assumed to represent the physiologically relevant similarities, while the large mode of larger distances represents random correlations. We fitted a mixture model of two Gaussians to the distribution and defined the threshold as the point where the wide left distribution becomes more probable. The same procedure was used separately for child and adult data.

The time courses and spatial topographies of each cluster were averaged to produce group level components. The criteria for the clusters of interest to be selected for further analysis were: 1) the cluster represented more than two-third of the participants and 2) the averaged cluster encompassed a clear stimulus related activity (difference between baseline and stimulus). Component separation was performed to unaveraged signal, so multiple component and component clusters had very little time locked activity. We evaluated the clusters manually to select the relevant clusters. Because of the sign ambiguity of the ICA method, the time courses were flipped to the better correlating direction. The ≤10-ms time shifts that were determined in the similarity computation were applied prior to averaging to diminish individual variation.

For comparison purposes, we compared the cluster averages to grand average time courses determined using more conventional region of interest (ROI) time-course extraction methods. We defined anatomical areas roughly corresponding to the cluster locations and used the first singular vector (PCA) method to extract the primary time course of the region. The ROIs were picked from the common Destrieux cortical parcellation (Destrieux et al. 2010). The ROIs were chosen to approximately cover the locations of the main clusters.

### Computing the Gating Ratio

We computed the MEG-response amplitude to the first and second stimulus as the total range of signal (i.e., peak-to-peak amplitude; see Fig. 2) similarly to Lenz et al. (2012). The same latency as after the first response was applied to the second response. Gating ratio was then defined as the ratio between the second and first stimulus peak-to-peak responses. The gating ratio was computed separately for each component-time course within each cluster. This approach was selected because it is feasible for short the ISIs, where the baseline activity level can vary between the successive stimuli. We used Welch’s *t*-test to compare gating values between child and adult clusters and Pearson’s correlation tests to look for age correlation.

## Results

We excluded five subjects from the child population due to noisy MEG signals or other problems during measurement. Typical reason was excessive muscle activity and head movement during MEG recording. All adult participants had acceptable data quality. All accepted participants encompassed a minimum of 90 epochs (i.e., successful stimuli) of which exactly 90 were used in the analysis. Accelerometer measurements indicated that the stimulus kinematics were stable between subjects. Standard deviation of movement timing was less than 1 ms and the peak acceleration varied only ~5%. The variation was most likely due to small intersubject variation in the accelerometer placement rather than actual differences in actuator speed. Standard deviation of actuator top speed estimated from the accelerometer was 0.17 cm/s. The stimulus kinematics are presented in Figure 1.

Average latency from proprioceptive stimulus onset (i.e., from movement onset) to measurable stimulus locked cortical activity was 30 ± 12 ms, that is, comparable to previously reported neural conduction time for the median nerve stimulation (Lueders et al. 1983; Dinner et al. 1987). It should be noted that although the onset of the evoked movements is very accurate (ms accuracy, Piitulainen et al. 2015), the actual stimulus to the proprioceptors is less punctuate than for electric median nerve stimulation due to the viscoelastic properties of the soft tissues, tendon, and muscles. For all the reported evoked cortical activities, the peripheral conduction delay was compensated so that the cortical response onset is always at zero time.

### Consistent Response Clusters in the Children

The clustering and post hoc selection of representative clusters resulted in 22 component clusters that included 18–24 components out of the 25 individual participants in each cluster. No cluster was full in the sense that it included a component from each of the 25 participants. The 22 clusters were reasonably well clustered with an average silhouette width of 0.85 (1 = perfect clustering).

**Figure 3:**
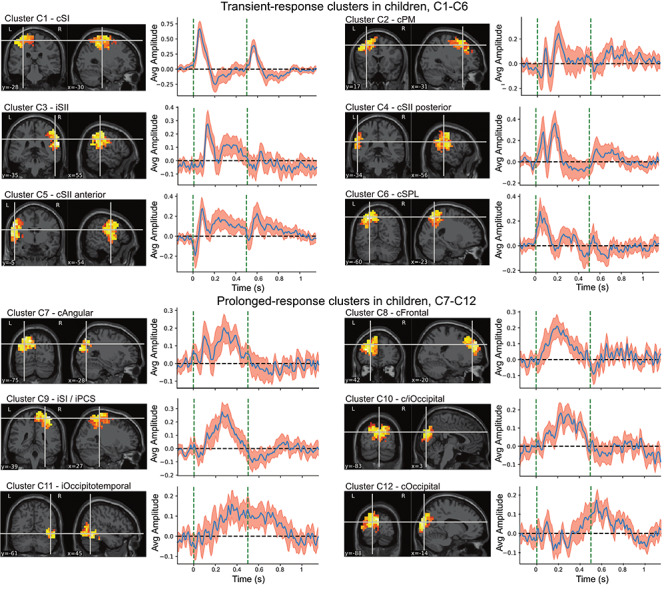
Twelve most consistent response clusters to the paired-proprioceptive stimulus in children. Spatial distributions are normalized cluster averages thresholded to 60% of the maximum. Cluster average timeseries are shown with a 95% confidence interval for the mean (in red). The dashed vertical lines indicate the onsets of the evoked responses at 0 and 0.5 s. Cluster averages are divided into transient responses that react to both stimuli and prolonged responses that only have one clear response in the stimulus interval.

We examined the clusters manually and excluded 10 clusters that showed no clear change after prestimulus baseline. The remaining 12 cluster averages were best defined both spatially and temporally within the somatosensory and motor cortices but were also detected in more anterior and posterior cortices. Figure 3 illustrates the response clusters for the children. Clusters C1–C6 are showing clear transient response to the proprioceptive stimulus. Clusters C7–C12 have more prolonged response shapes. Spatial distributions of the cluster means are thresholded to 60% of the peak in the visualizations, the choice being arbitrary, but the distributions seem focal with a clear maximum. MNE-based inverse solutions for each true source span the entire source space; thus, the approximate location of the peak is more relevant than the spatial extent of the distribution.

The cluster representing the primary proprioceptive response in the SI cortex (cluster C1, Fig. 3) shows a clear transient peak and small negative deflection after both stimuli. Activity related to the return of the finger (flexion) to the initial position at 200 ms after the first peak was not clearly identifiable. This was expected as the movement stimuli were deliberately designed to induce a sharp extension (the primary stimulus) and slower flexion (return) to reduce confounding stimulus effects between the repeated stimuli. Cluster C1 in the SI cortex contralateral to the stimulus corresponded well with the grand-average timeseries from postcentral gyrus (Supplementary Fig. 1).

Clusters C2 and C4 show very similar temporal characteristics in the contralateral premotor cortex, and posterior lateral sulcus areas (Fig. 3). These responses were characterized by a dual peak after the first stimulus (before 200 ms) and a substantial decrease (gating) in amplitude after the second stimulus. The dual peak characteristics are also visible in grand average timeseries from subcentral gyrus (Supplementary Fig. 1).

Both clusters C3 and C5 show a delayed transient peak followed with a more sustained response in the contra and ipsilateral lateral sulci, respectively. This could represent expected bilateral SII cortex activation to the somatosensory stimulus, as demonstrated earlier using MEG for tactile (Hari et al. 1993) and proprioceptive (Alary et al. 2002) stimuli. The contralateral response included an early peak concurrent with the primary somatosensory response that was lacking from the ipsilateral responses. Ipsilateral peaks were also delayed by 50 ms compared with contralateral ones. Cluster C6 peaked at the superior parietal lobule (Brodmann’s areas 5 and 7) and showed two distinct peaks at roughly 200-ms intervals with strong gating effect.

Clusters C7–C12 represent delayed and prolonged responses that were located in the contralateral frontal (cluster C8) and occipital (clusters C7 and C12) cortices, ipsilateral parietal (cluster C9) and occipital (cluster C11) cortices, and bilateral occipital cortices (cluster C10). Despite very different spatial representations, the clusters C8–C10 show very similar temporal characteristics peaking around 200 ms.

### Consistent Response Clusters in Adults

**Figure 4:**
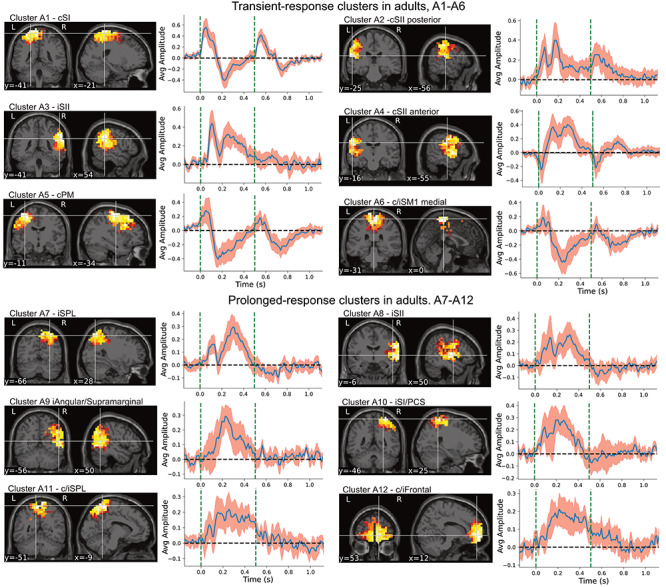
Twelve most consistent response clusters to the paired-proprioceptive stimulus in adults. Spatial distributions are normalized cluster averages thresholded to 60% of the maximum. Cluster average timeseries are shown with a 95% confidence interval for the mean (in red). The dashed vertical lines indicate the onsets of the evoked responses at 0 and 0.5 s. Cluster averages are divided into transient responses that react to both stimuli and prolonged responses that only have one clear response in the stimulus interval.

In line with child data analysis, we chose the same number of 12 most representative clusters from the adult data for physiological comparison and methodological validation purposes. Figure 4 illustrates the response clusters for the adults. The only clear consistent difference compared with the children is the stronger negative rebound after the initial peak and is visible in multiple clusters (e.g., clusters A1, A5, and A6).

Clusters C3 and A3, from child and adult data, respectively, correspond to ipsilateral lateral sulcus (SII cortex) response with very similar average time course for both groups. Clusters C4 and C5 and A2 and A4, corresponding to contralateral lateral sulcus responses, in child and adult data, respectively, also appear very similar although in the adults show more prominent activity after ∼200 ms.

Likewise, to the children, there are several clusters (clusters A7–A12, Fig. 4) showing a slow response after the first stimulus and very limited response to the second one. These were located in frontal and parietal cortices. The ipsilateral SI response appeared very similar in both adults and children (clusters C9 and A10, respectively).

### Gating Ratios in Children and Adults

The early M1 and SI cortex responses contralateral to the movement appeared similar for both groups (Figs 3 and 4). Table 1 shows the group average gating ratios for the six transient response clusters in children and adults. The gating effect was statistically significant in each cluster. Interindividual variability was high compared with between cluster differences. Gating ratio was not computed for the prolonged response clusters as they showed a negligible response to the second stimulus. The gating ratio of the primary SI cortex cluster contralateral to the stimulus did not differ between the children and adults (i.e., clusters C1 and A1; Welch’s *t*(35.22) = 1.04, *P* = 0.31). In addition, no significant differences in gating ratios between the groups were found for the remaining clusters detectable for both groups in the posterior (Clusters C4 and A2: Welch’s *t*(29.4) = −1.02, *P* = 0.32) or anterior (C5 to A4: Welch’s *t*(30.0) = 0.68, *P* = 0.50) parts of the contralateral SII cortex or ipsilateral SII cortex (C3 and A3: Welch’s *t*(22.3) = −1.00, *P* = 0.32). The participants’ age did not correlate with the gating ratios. Pearson correlation coefficient between age and gating ratio within SI cortex clusters for children (C1) was *r*(17) = 0.07, *P* = 0.78 and for adults (A1) was *r*(17) = −0.07, *P* = 0.77. When the children (C1) and adult (A1) clusters were combined, the correlation between age and gating ratio was nonsignificant (*r*(36) = 0.06, *P* = 0.74). The intersubject variation of the gating ratio was high compared with expected effect size.

**Table 1:** Mean (±SD) gating ratios for the transient clusters in children and adults

Cluster	Gating	*t*	*P*
Children			
Cluster C1 (cSI)	0.72 ± 0.22	5.27	<0.01
Cluster C2 (cPM)	0.72 ± 0.23	4.59	<0.01
Cluster C3 (iSII)	0.73 ± 0.31	3.60	<0.01
Cluster C4 (cSII)	0.61 ± 0.17	8.31	<0.01
Cluster C5 (cSII)	0.76 ± 0.22	4.16	<0.01
Cluster C6 (cSPL)	0.87 ± 0.30	2.61	<0.05
Adults			
Cluster A1 (cSI)	0.69 ± 0.18	4.9	<0.01
Cluster A2 (cSII)	0.62 ± 0.23	5.6	<0.01
Cluster A3 (iSII)	0.52 ± 0.23	5.9	<0.01
Cluster A4 (cSII)	0.65 ± 0.18	6.21	<0.01
Cluster A5 (cPM)	0.69 ± 0.21	6.18	<0.01
Cluster A6 (c/iSM1)	0.61 ± 0.19	5.01	<0.01

*Note:* The *t*- and *P*-values are from a paired sample *t*-test between amplitude for the first and second stimulus.

## Discussion

Our spatial ICA and clustering approach extracted consistent cortical activity patterns for paired-proprioceptive stimuli of the index finger. Each cluster represented independent components of the cortical processing related to the evoked proprioceptive afference. The gating ratio of the primary proprioceptive response in the SI cortex (represented by the first cluster) was comparable to the earlier observations using other somatosensory stimuli (e.g., Spooner et al. 2019). The participants’ age did not correlate with the gating ratio and was at similar level in children and adults. Thus, the mechanisms behind gating appeared to be primarily matured in the 10- to 17-year-old participants; however, some differences were observed to adults in more delayed proprioceptive processing after ≥150 ms poststimulus. Proprioceptive stimulation activated the cortex broadly beyond the SM1 cortex, and the gating ratio was similar across the specific cortices. The proprioceptive gating appears to be comprehensive in nature throughout the human brain. These results indicated that the spatial ICA source separation-based approach is feasible tool to extract extended neurophysiologically relevant information beyond the strongest primary cortical responses to proprioceptive and likely also to other sensory simulations.

### Proprioceptive Gating in Children and Adults

The primary aim of this study was to decompose the proprioceptive gating to distinct cortical components within and beyond SI cortex using a novel method. Our method did indeed reveal new important insights about cortical proprioceptive processing in children and adults. Most previous studies in the somatosensory domain have focused on the somatosensory gating of the most prominent response arising from the SI cortex. To our knowledge, there are no prior studies investigating the effect of age on gating in children and adolescents.

The proprioceptive gating ratio in the SI cortex was at similar level as reported earlier for mechanical tactile and electrical peripheral nerve stimulations (Edgar et al. 2005; Thoma et al. 2007; Wiesman et al. 2017). The gating ratio for the early proprioceptive processing was at similar level in children and adults, indicating that the proprioceptive system is primarily matured before or shortly after the start of puberty, or even earlier. Furthermore, the participants’ age did not significantly correlate with the gating ratio in any of the clustered components within our group of 10- to 17-year-old children and adolescents. In the other spectrum of the lifespan, the somatosensory gating has been found to be reduced in older individuals (i.e., over 50 years old) corresponding to decline in tactile discrimination performance (Lenz et al. 2012; Cheng and Lin 2013; Spooner et al. 2019). In addition, the cortical proprioceptive processing has shown to be altered in the older individuals being associated with worse postural standing balance performance (Piitulainen, Seipäjärvi, et al. 2018b). However, gating experiment in this domain is lacking.

The only consistent difference between adults and children in the transient responses, including the primary response (C1 and A1), was the visibly larger activity at 200 ms in the adults. This activity may reflect more complex proprioceptive processing in a wider cortical neuronal network. Although the physiological mechanisms are unclear, it could be that the development of this network is slower compared with the early proprioceptive processing in the SI cortex.

### Spatiotemporal Properties of Proprioceptive Gating

We demonstrated that proprioceptive gating occurs within a large cortical network, including SI (BA3, 1, 2) and SII cortices, premotor cortex (BA6), and superior parietal lobule (BA5 and 7). These regions are all relevant for sensorimotor processing and integration. Furthermore, we found a widespread cortical neuronal network of prolonged responses that demonstrated very strong gating or were nonresponsive to the second stimulus.

*The transient-response clusters* that presented clear peaks after both stimuli (C1–C6 and A1–A6) showed consistent gating behavior. The C1 cluster in children represented the expected primary response to the proprioceptive stimulation in the SI cortex, and a corresponding cluster (A1) in adults appeared very similar but exhibited the larger rebound after the initial peak.

Cluster C2 represented most likely premotor activity. The cluster was spatially widespread, and its exact peak response location was hard to define. This activity could originate from the ventral premotor cortex which has been found to have a role in hand movement and object manipulation (Binkofski et al. 1999; Binkofski and Buccino 2006; Miquée et al. 2008). Spatially, the cluster C2 corresponded approximately to cluster A5 in the adults, but the temporal characteristics of the activities were different.

We also found consistent responses bilaterally in the lateral sulci, most likely representing proprioceptive processing in the SII cortices. The response in the ipsilateral posterior lateral sulcus (C3 and A3) peaked at 150 ms after the response onset. These clusters were remarkably similar both spatially and temporally between the children and adults, suggesting that the neural source is consistent between the populations. For the contralateral SII cortex, two spatially distinct clusters were detected both in adults and children (C4, C5, A2, and A4). The more posterior cluster (C4 and A2) indicated a dual-peaked initial response with ∼100-ms interval. The more anterior cluster (C5 and A4) demonstrated a small initial negative peak followed with large positive deflection. The deflection was visibly more prominent in adults than children especially after 250 ms. Previous work has suggested that the somatosensory M200 component of tactile stimulation is localized to SII cortex (Nevalainen et al. 2008), whereas based on our observation, the activity in the same time frame was spread to multiple cortical regions beyond the SI and SII cortices.

*The delayed and prolonged response clusters* were found in frontal and parietal lobes both in children and adults (C7–12 and A7–12). These responses continued beyond the onset of the second stimulus (onset at 0*.*5 s) and reacted negligibly if at all to the second stimulus. This could indicate that activity in these regions is associated more to the higher level processing or integration of the proprioceptive afference, and not the early somatosensory processing. In this context, a complex bilateral network connecting the frontal and parietal cortices in somatosensory processing could be involved. These fronto-parietal networks (FPNs) are, for example, linked to attention control (Coull et al. 1996; Wang, Liu, et al. 2010), which might be relevant also for gating of excessive sensory afference. Likewise, abnormal gating (Edgar et al. 2005; Thoma et al. 2007) and reduced function in FPN have also been linked to schizophrenia (Roiser et al. 2013). However, the role of FPN in sensory and especially in proprioceptive gating still requires further studies to be confirmed.

In children, there were two clusters in the occipital areas (clusters C10 and C12). It is possible that these clusters primarily reflect slightly mislocalized posterior parietal activity similar to clusters C7, A7, A9, and A11. It is unlikely that the visual system would have been activated systematically by the proprioceptive stimuli. Cluster C11 reflects occipitotemporal activity which seems unintuitive for the proprioceptive stimulation. It is possible that this activity is projected from deeper sources not well visible using MNE-based inverse methods. Some activity might also be projected from the cerebellum, which was not included in the source model. Cerebellum receives fast proprioceptive input, and MEG responses to finger movement stimulus has been demonstrated previously (Marty et al. 2018).

### Paired-Proprioceptive Stimuli—Strengths and Weaknesses

There are several challenges when examining sensory gating in the proprioceptive domain using natural stimuli. The Paired-movement stimuli with short intervals are challenging because the finger needs to be returned to the initial position prior the next stimulus. This can cause the contamination of the primary stimulus response or baseline of interest by the returning movement. However, we concluded that the contamination effect to the second primary response was negligible, most likely because we kept the applied movement range short enough (∼6 mm). The shape of the main response, that is, SI cortex response cluster, was similar to earlier reported responses using the same proprioceptive stimulator for index finger stimulation (Smeds et al. 2017). Smeds et al. (2017) used equivalent current dipole modeling to extract the evoked-response strength in SI cortex and showed that with short below 0.5-s ISI, the response would be strongly reduced. This strong gating effect may explain why we did not observe a clear response for the returning finger-flexion movement that occurred immediately after the finger extension. Only in cluster C6 in the superior parietal lobule (BA 5 and 6), it was possible to detect also a response pattern coinciding with the returning movement. The superior parietal lobule has important role in the sensory integration including proprioception-based limb orientation and position (Felician et al. 2004; Limanowski and Blankenburg 2016). This may explain its sensitivity to fine proprioceptive details of the hand and detecting the fine kinematics of the current extension-flexion movement.

It should be noted that the pneumatic muscle actuator did not return completely to the initial position between the paired stimuli (see Fig. 1). This was inevitable, because we attempted to keep the returning flexion movement slower than the actual stimulus (fast extension movement). The properties of the pneumatic artificial muscle did not allow full return to the initial position in ~250 ms. However, the speed of the actual paired stimuli was similar. The speed is likely more crucial factor compared with the ~2-mm difference in the initial position of the finger because the proprioceptors are activated by the movement rather than exact position (Proske and Gandevia 2012).

The current evoked finger movements activate primarily the proprioceptors; however, some skin movement and tension around the metacarpophalangeal joint and tip of the finger is inevitable that may activate some of the cutaneous mechanoreceptors. We attempted to minimize tactile afference by taping the fingertip with two layers of surgical tape and attaching the finger firmly to the pneumatic actuator so that the tactile sensation was kept constant. In addition, it has been shown that when using MEG, the cortical responses to passive movements reflect primarily the proprioceptive afference (Piitulainen et al. 2013). Some of the cutaneous receptors may be considered as proprioceptors as their functions are partly overlapping, and thus have an important role in proprioception. Therefore, some overlap in the cortical patterns is inevitable (Collins et al. 2000).

However, there is a minute pressure change in the fingertip during the actuator movement, and its exact effect to the conclusions of this study is difficult to quantify due to overlap between respective cortical processes. Tactile receptors in the skin also play an important role in proprioception, so some overlap in the cortical patterns is inevitable (Collins et al. 2000).

Attention is known to have an effect in cortical gating. In the current study, participants’ attention to the proprioceptive stimuli was not quantified. However, we do not expect strong systematic effect of attention for the current results. Furthermore, the randomized stimulus order and jittered timings of the stimuli most likely mitigated the possible effect. Additionally, it has been shown, for example, that movie watching does not influence somatosensory responses (Espenhahn et al. 2020). We instructed the participants to focus on an uneventful video to help them to direct their attention away from the stimuli.

It should be noted that the interindividual variability in the gating ratio was high. Therefore, it is difficult to draw reliable conclusions at the individual level or using small sample sizes. The currently used proprioceptive stimuli and the related MEG responses have proved to be very reproducible at the group level, even with 1-year follow-up (Piitulainen, Illman, et al. 2018a). However, caution needs to be taken if the aim is to follow individuals, such as a patient. Reproducibility of the proprioceptive gating in repeated sessions has not been examined. In the auditory domain, the reproducibility depends on the method used for computing the amplitude and gating values (Fuerst et al. 2007; Rentzsch et al. 2008). In addition, the different response components show different reproducibility. Interindividual variability of the gating ratio has been high, and the patient and healthy control populations overlap strongly even though there are statistically significant difference between the populations (Patterson et al. 2008). Although our proprioceptive stimuli were very stable, we cannot entirely rule out the effects of interindividual variations in the proprioceptive stimulation, for example, in hand position or possible tactile interference. However, such small differences do not likely explain the high interindividual variation that has been detected for all sensory domains.

### Spatial ICA—Strengths and Weaknesses

ICA-based approach enables the extraction of weaker cortical activities from MEG signals that might go undetected if using more conventional methods. The weaker activities are often unobtainable since they are covered by sensor or brain noise sources or a strong primary activity extended by the point spread of the inverse solution. We validated this problem by extracting grand average timeseries from multiple ROIs corresponding to the cluster locations (Supplementary Figs 1 and 2). We found highly correlated early peak activity visible in most regions. This is likely just a point spread from the primary response location. However, the primary concern with ICA methods is the difficulty to interpret the results. FastICA algorithm used in the current study acts essentially as a “black box,” and thus, it is hard to discern what are the exact causes for a particular component separation. Furthermore, FastICA, as a fixed-point iteration-based algorithm, is inherently unable to indicate whether global optimal solution is found (Hyvärinen and Oja 1997). There are possible methods to mitigate this problem (e.g., Himberg et al. 2004; Du et al. 2014). However, this problem is less significant in MEG compared with fMRI analysis, because of the better MEG signal to noise ratio compared with fMRI resting state recordings. For the current MEG data, successive test runs of the ICA with randomized initialization produced very consistent results.

Spatial ICA is frequently used in fMRI analysis but less in MEG, probably because MEG-based ICA analysis has largely concentrated on sensor space, and the number of sensors provides few sample points for ICA. Also combining different information modalities, such as magnetometers and gradiometers, can be problematic in sensor space. In addition, the MEG signals in sensor space are highly spatially overlapping, and thus, their meaningful separation is more challenging compared with the source space. In the source space, the inverse model has already spatially filtered the activity from the sensors to the cortical locations. The results are also simpler to justify physically in the cortical space compared with the sensor space that possess multiple factors affecting how each source is seen by a given sensor.

Using spatial ICA over temporal one is justified because temporal ICA would assume that the time locked responses from different cortical regions are temporally independent. We did tests using temporal ICA and validated that it tends to combine temporally correlated cortical regions in the somatosensory areas under single spatially wide component. Spatial ICA on the other hand does not require temporal independence. In recent years, spatial ICA has been applied to Hilbert envelopes derived from cortical oscillatory resting state activity (Ramkumar et al. 2014), but to our knowledge, there has not been attempts to apply spatial ICA to evoked responses. For the proprioceptive evoked MEG data, the spatial ICA appears to find primarily relatively focal distributions, potentially implying highly localized approximately dipolar sources in these cortical locations. However, it is challenging to demonstrate that this is the case. It is likely that the ICA process cannot always perfectly separate the sources, meaning that the resulting components can still be a mixed combination of the true sources. We cannot assume that a true separation of the original sources is achievable without the ground truth.

The properties of the distributed inverse solution can affect the separation significantly. In the current study, we used MNE-based dSPM method (Dale et al. 2000) because we thought it would be neutral about the spatiotemporal properties of the response while offering good dipole location accuracy (for assessment of MNE methods, see Lin et al. 2006; Hauk et al. 2011). In comparison, beamformers assume uncorrelated sources in the first place. This affects the independent component separation compared with minimum norm solutions because possible correlated sources are already attenuated by the beamforming. Another issue is localization errors and biases generated by the source estimation methods. These errors may shift the cortical activity distributions and consequently hinder the correct separation of the respective components. Localization errors are partially dependent on the specific individual factors, such as the accuracy of the cortical model, quality of MEG recording, and co-registration and interindividual variability in the functional anatomy. Altogether, this means that the component corresponding to the same functional activity may localize slightly differently across the individuals. We assume that these errors were insignificant in the current data. Nevertheless, it is currently very challenging to meaningfully validate the ICA decomposition results using physiological data.

It should also be noted that we did not study the inverse solutions for magnetometers or gradiometers separately but for their combination. Due to their technical differences, gradiometers and magnetometers measure partly different features of the cortical signal, which can be considered an advantage, but have different units and sensitivity scale. The use of different sensor types may, in some circumstances, distort the resulting source estimate. To overcome this issue, use of prestimulus (i.e., baseline) noise covariance for the regularization term in the minimum-norm estimation effectively spatially whitens the data and converts the magnetometers and gradiometers to the same base. In addition, tSSS denoising algorithm that we applied significantly diminishes the spatial differences of source topographies estimated with magnetometers and gradiometers (Garcés et al. 2017). Taken together, we do not expect the choice of using both magnetometers and gradiometers to have significant impact on the reported results.

Despite the methodological limitations, the spatial ICA detected several consistent separate spatially distinct components that were challenging to distinguish from simple averaged evoked responses. The detected components corresponded well with the main features of the grand average time courses. Spatial ICA is a fully data driven method and thus does not require prior knowledge or model of the response. Therefore, it shows a high potential as a tool for exploratory MEG studies, as well as for generating new research hypotheses.

### Clustering—Strengths and Weaknesses

In the current study, we chose to first run individual ICA, and then cluster the extracted components to derive group level results. Another common approach is to concatenate the data across all individuals and perform dimensionality reduction and run ICA to the resulting group-data matrix to derive the group results. The latter approach is simpler and allows a trivial back projection to check how each individual contributes to each component. However, as the data are concatenated in the group ICA, it assumes that the individuals share the same mixing matrix. That is, no anatomical, functional, or method derived variation between the individuals. For this reason, each component derived by the group ICA includes only the “common” part of each individual’s data that can be explained by the generalized group component. The currently applied clustering of individual ICA components allows more interindividual variability in the functional anatomy of the brain, because the components are not required to be identical across the individuals. This is beneficial feature as it is well known that interindividual variability is inevitable between the brains.

Since the ICA was performed to unaveraged data, featuring activity that is not stimulus locked, multiple components had activity that diminished during averaging. While we reduced several least relevant components from each subject, we were conservative with this as we did not want to lose information. These components feature spurious correlations that created clusters with very little stimulus-related activity. We picked most relevant clusters manually to mitigate this, but it would be beneficial if this could be automated in a data driven manner in the future.

The primary issue with the clustering approach, at least in the current study, was that the clustering result is sensitive to the chosen methods and parameters. For example, adjustment of the clustering threshold or weighting of spatial and temporal similarity may change the features of the clusters significantly. To mitigate this issue, we used a data driven approach to define the clustering threshold. The applied clustering algorithm only allowed to pick one ICA component from each individual subject to each cluster. The hierarchical clustering algorithm combines neighboring clusters according to distance criterion, and if two clusters contain a component from the same individual, the clusters cannot be combined. However, some subjects had multiple ICA components that were relatively close to each other and probably should have been included in the same cluster to avoid the generation of multiple clusters from the same physiological activity. It might thus be beneficial to choose a further reduced number of sufficiently different components form each individual for the analysis. The current child and adult data sets had reasonable similarity in the resulting clusters. This is a promising result about reliability and robustness of the method and its future use.

We used an arbitrary threshold of two-third of participants for a cluster to be considered group representative. Ideally each cluster would have contained a component from each subject, but this was not practically possible due to the variation of source estimation and ICA decomposition results. A data driven method should be developed to decide which clusters to choose as representative. However, we think that the results using this simple threshold were reasonable.

### Future Prospects

Spatial ICA clustering methodology could potentially be used to examine populations with neuronal disorders. The brain lesions, tumors, and other prominent structural deformations are a major challenge when examining the affected brain function in the group level. The currently used correlation similarity metric requires the individual cortices to be morphed to a common reference brain and thus is not suitable for patients with major deformities. To overcome this issue, a different metric could be used without morphing, for example, similarity based on the anatomical peak location of the component. Such modifications could further extend the feasibility of the proposed methods to patient populations by allowing more interindividual anatomical variation.

The current experimental design was not aimed for studying induced responses, for example, in the rolandic rhythms but the gating effect to the paired stimulus. However, such induced responses could be linked to the gating behavior and especially to some of the currently observed more delayed and slower response clusters. ICA has been previously used to examine the induced responses in frequency and spatial domains (Hyvärinen et al. 2010; Ramkumar et al. 2014).

Gating has been shown to be involved in attention control (e.g., Cheng et al. 2016), but associations and causality between attention-based gating, paired-stimulus paradigms, and the respective brain networks are not well established. Therefore, it may be that selective-attention task during somatosensory stimulation may alter the functional state of the frontal and parietal response components, and this may further modulate somatosensory gating in the SI cortex. According to our observations of fronto-parietal response clusters, it is possible that the same cortical network is involved in the gating of repeated proprioceptive stimuli.

## Conclusion

In this study, we applied spatial ICA and clustering of components in the source space to proprioceptive MEG responses of the hand in children and adults. Consistent transient cluster corresponding to primary SI cortex response was detected and was accompanied with a variety of transient clusters beyond the SI cortex, all exhibiting gating behavior in very similar manner. The gating ratio for proprioception was around 70% in line with previous findings in other sensory domains. We did not find age-related differences in gating of the primary responses. The only visible difference was more prominent proprioceptive processing at ≥150 ms poststimulus in the adults. Therefore, it is possible that some aspects of the processing of proprioceptive stimuli in the brain mature still in the early adulthood. Finally, we found delayed and prolonged activity components in the middle frontal areas and posterior parietal areas, which likely corresponded to more higher order sensorimotor processing. These components did not visibly react to the second stimulus.

## Notes

We thank technical support from Helge Kainulainen in building the pneumatic-movement actuators at Aalto NeuroImaging, Aalto University, Espoo, Finland. The calculations presented above were performed using computer resources within the Aalto University School of Science “Science-IT” project. *Conflict of interest:* None declared.

## Funding

Academy of Finland (grants nos 296240, 307250, 304294, 326988, 327288); Jane and Aatos Erkko Foundation to H.P.

## Supplementary Material

FigureS1_color_bhaa306

FigureS2_color_bhaa306

Supplementary_Figure_Legends_bhaa306
