# Supplementary figures and images for: Gating Patterns to Proprioceptive Stimulation in Various Cortical Areas: An MEG Study in Children and Adults using Spatial ICA
Source: Cereb Cortex. 2020 Nov 3;31(3):1523–37. doi: 10.1093/cercor/bhaa306 (PMC7869097; doi:10.1093/cercor/bhaa306)

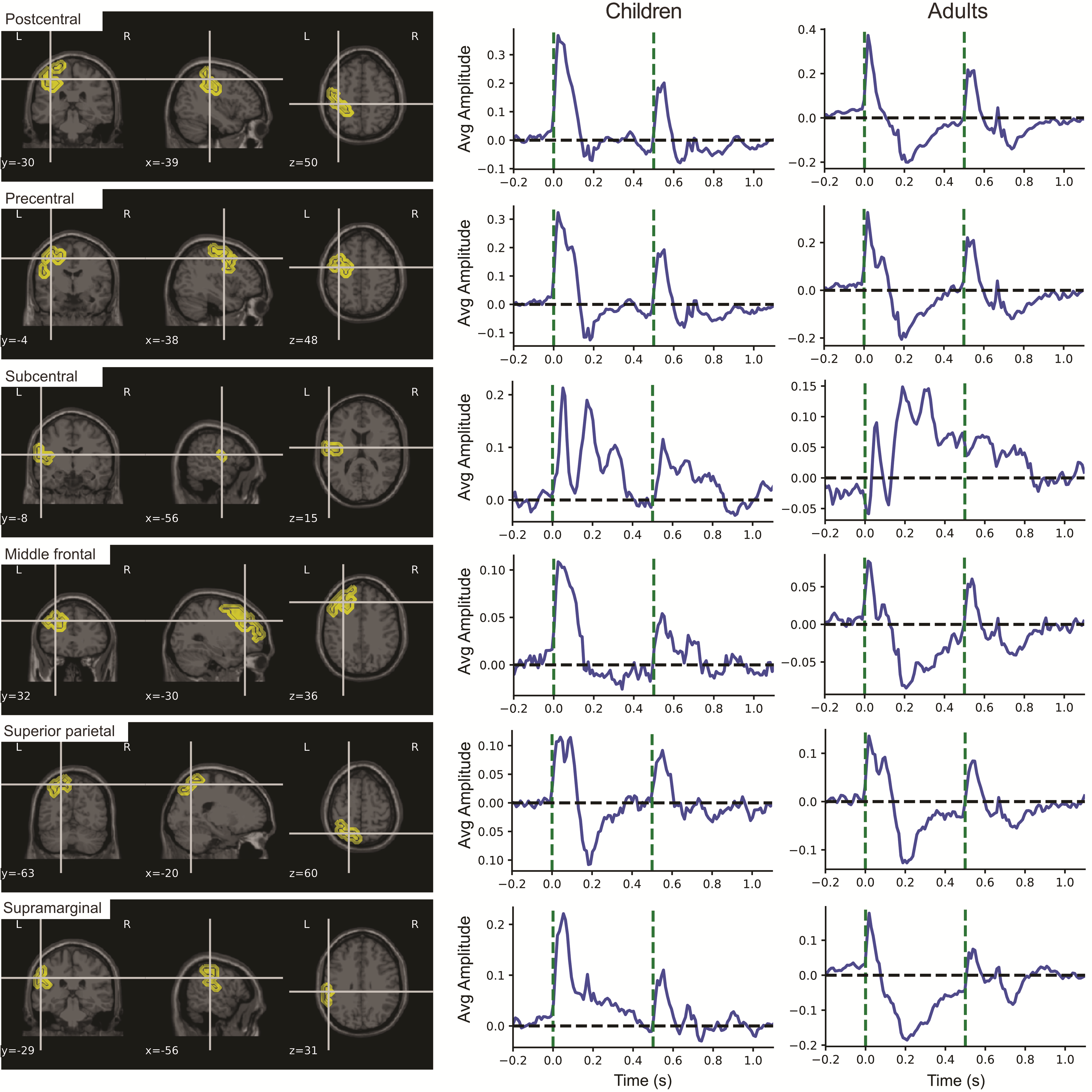

Supplement: FigureS1_color_bhaa306 [file figures1_color_bhaa306.png]

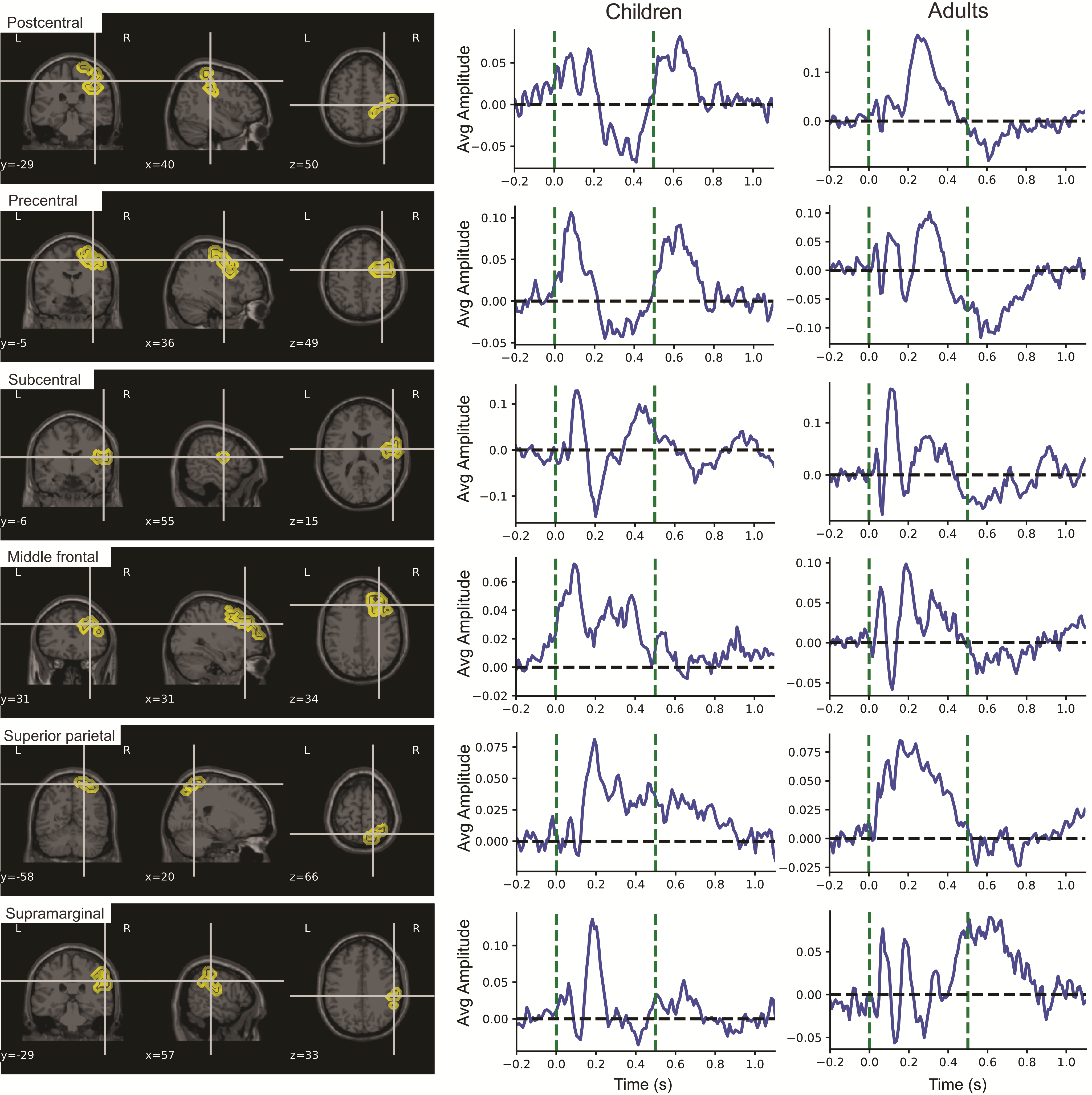

Supplement: FigureS2_color_bhaa306 [file figures2_color_bhaa306.png]
