# Supplementary material for: Gating Patterns to Proprioceptive Stimulation in Various Cortical Areas: An MEG Study in Children and Adults using Spatial ICA
Source: Cereb Cortex. 2020 Nov 3;31(3):1523–37. doi: 10.1093/cercor/bhaa306 (PMC7869097; doi:10.1093/cercor/bhaa306)
Supplement: Supplementary_Figure_Legends_bhaa306 [file supplementary_figure_legends_bhaa306.docx]

Figure S1: Grand average responses for anatomical ROIs (Destrieux et al. 2010) contralateral to the proprioceptive stimulation. Left: ROI shown in yellow. Right: The first singular vector of the grand average response in each ROI for the child and adult participants respectively. The dashed vertical lines indicate the onsets of the evoked responses at 0 and 0.5 s.

Figure S2: Grand average responses for anatomical ROIs (Destrieux et al. 2010) ipsilateral to the proprioceptive stimulation. Left: ROI shown in yellow. Right: The first singular vector of the grand average response in each ROI for the child and adult participants respectively. The dashed vertical lines indicate the onsets of the evoked responses at 0 and 0.5 s.
